# Correlates of sun protection behaviors among melanoma survivors

**DOI:** 10.1186/s12889-021-10951-1

**Published:** 2021-05-08

**Authors:** Carolyn J. Heckman, Sharon L. Manne, Deborah A. Kashy, Trishnee Bhurosy, Lee Ritterband, Elliot J. Coups

**Affiliations:** 1 Rutgers, The State University of New Jersey, 195 Little Albany St., New Brunswick, NJ 08901 USA; 2 Michigan State University, East Lansing, MI USA; 3 University of Virginia, Charlottesville, VA USA

**Keywords:** Melanoma survivors, Sun protection behaviors, Prevention, Skin cancer, Melanoma recurrence

## Abstract

**Background:**

The study objective was to assess potential correlates of sun protection behaviors among melanoma survivors.

**Methods:**

Participants were 441 melanoma survivors recruited from three health centers and a state cancer registry in the United States. Sun protection behaviors (sunscreen, shade, protective shirts, and hats) were assessed through an online survey, as were potential correlates (demographic, melanoma risk, knowledge and beliefs, psychological and social influence factors). Hierarchical multiple regression analyses were conducted.

**Results:**

Correlates of sun protection behaviors included education, skin cancer risk factors, melanoma knowledge and beliefs, melanoma worry and distress, physician recommendation for sun protection, injunctive norms, and pro-protection beliefs (e.g., perceived barriers, self-efficacy).

**Conclusions:**

Future efforts to improve sun safety among melanoma survivors may benefit from targeting individuals with lower education levels, and addressing sun protection social influence, barriers, and self-efficacy.

## Background

With recent improvements in diagnosis and treatment, the number of individuals surviving after a melanoma diagnosis has been increasing [1]. There are more than 1.2 million melanoma survivors in the United States [2]. Unfortunately, these individuals are at elevated risk for melanoma recurrence, basal and squamous cell cancers, and second primary cancers [1]. Ultraviolet radiation from the sun is a contributing factor in the vast majority of melanomas and other skin cancers [3]. Thus, in addition to ongoing skin cancer examinations by a healthcare provider and regular skin self-examination, engaging in regular sun protection behaviors is recommended for melanoma survivors in order to reduce their risk of subsequent skin cancers [4].

Although melanoma survivors engage in more sun protection behaviors than the general population [5, 6], engagement is suboptimal [7]. Between 15 and 43% of melanoma survivors report sunbathing [8–10], and between 2 and 6% report indoor tanning [5, 8]. Engagement in sun protection behaviors is low (7–67% never engage in one or more sun protection behavior) and is very low with regard to wearing sun protective clothing such as a wide-brimmed hat (67% never wear) or long-sleeved shirt (41% never wear) as well as avoiding midday sun (55% never avoid) [5, 7, 11].

Understanding demographic and psychosocial factors associated with sun protection behaviors among melanoma survivors can assist in focusing more intensive intervention efforts on those less adherent survivors, as well as identify possible intervention targets. The limited research suggests that older age, female sex, greater education, and higher self-efficacy are associated with greater overall sun protection behaviors [4, 11–13]. Additionally, Manne and Lessin [12] found that higher levels of sun protection behaviors were associated with receipt of regular dental care (a proxy for access to healthcare), as well as greater melanoma knowledge, physician recommendation for sun protection, perceived sun protection descriptive norms, and lower advantages of sun exposure.

The aim of the current study was to expand the examination of potential correlates of sun protection behaviors to key factors that have not been explored. Selection of correlates was based on the Preventive Health Model [14], normative influences from the Theory of Planned Behavior [15], and prior research on factors related to sun protection and melanoma survivors [12, 16–23]. Correlates assessed included demographic factors, medical factors, skin cancer risk factors, melanoma knowledge and beliefs, psychological characteristics, social influence, and beliefs about sun protection behaviors (perceived benefits, perceived barriers, self-efficacy). Three new potential correlates were examined: history of sunburn, injunctive norms for sun protection behaviors, and perceived controllability of melanoma. Sunburn is an important correlate to consider, as it is a risk factor for melanoma and suggests inadequate sun protection behaviors [24]. Injunctive norms (beliefs about what behaviors significant others think one should perform) may be even more important than descriptive norms (beliefs about how others behave) in predicting behavior [25]. Third, greater perceived controllability has been found to be relevant for other cancer prevention behaviors and behavior change interventions [26]. The findings from this study could help improve understanding of potential predictors of sun protection behaviors and inform future sun safety interventions for melanoma survivors in terms of selecting important populations, behaviors, and psychosocial constructs to target.

## Method

### Overview

The study was conducted as an online baseline survey of a randomized controlled trial—the mySmartSkin web-based intervention designed to increase skin self-examination and sun protection behaviors among melanoma survivors. A detailed description of the mySmartSkin study, including recruitment, measures, and other analyses, is available elsewhere [27]. The Institutional Review Boards affiliated with the University and Medical Center approved this study.

### Recruitment

Participants were adult melanoma survivors who were recruited through four different sites within one US state: a National Cancer Institute-designated comprehensive cancer center; a Department of Dermatology; a Medical Center; and a State Cancer Registry. Potentially eligible participants received a study information letter and consent form, and a member of the research team attempted to contact each patient to determine their eligibility. For patients recruited through the NJSCR, an information letter was mailed to each patient’s physician requesting that the physician contact the research team within 2 weeks if there were any reasons that the patient should not be contacted for the study. The research team at the Cancer Institute determined the eligibility of patients who expressed interest to take part in the study. Of the 1411 individuals assessed for eligibility and study interest, 150 (10.6%) were deemed ineligible, and 776 (55%) declined to participate, with the most common reason provided being lack of interest (*n* = 399). Of 485 patients who consented to the study, 441 (90.9%) participants completed the online survey. Upon completion of the baseline survey, each participant received a $25 gift card.

### Eligibility criteria

Inclusion criteria to participate in the study were: a) diagnosed with primary pathologic stage 0-III cutaneous malignant melanoma; b) were 3 to 24 months post-surgical treatment; c) were not performing thorough SSE (i.e., they did not report inspecting each of 15 areas of the body at least once during the past 2 months) [28] and/or not following sun protection recommendations (i.e., a mean score of < 4.0 on a sun protection behavior index that assessed the frequency of engaging in four behaviors, each assessed on a 5-point scale from 1 = never to 5 = always [29]; d) were 18 years old and above; e) had Internet access; f) able to read and speak in English and g) able to provide informed consent.

### Measures

#### Outcomes

Four individual sun protection behaviors (use of sunscreen with sun protection factor [SPF] 30 or more, wearing a long-sleeved shirt, wearing a wide-brimmed hat, and staying in the shade or under an umbrella) were assessed over the past 12 months (e.g., “In the last 12 months, when you were outside on a sunny day, how often did you wear a long-sleeved shirt?”) (1 = *never* to 5 = *always*) [29]. The mean of these four items was calculated to create a total index of sun protection behaviors in the past 12 months.

#### Correlates

##### Demographics

Participants reported their age, education level, marital status, and sex.

##### Medical factors

Medical records were used to extract months since surgery and disease stage at diagnosis (stage 0, stage 1, stage 2, and stage 3).

##### Skin cancer risk factors

Participants reported the presence or absence of eight skin cancer risk factors, which resulted in a total score from 0 to 8: light eye color, light natural hair color, fair untanned skin, skin sensitivity, presence of freckles, presence of large moles, ever indoor tanning, and family history of melanoma [12, 19]. Participants also reported how many times they had experienced a “red, pink, or painful sunburn that lasted a day or more” in the last year, which was dichotomized into zero versus one or more sunburns.

##### Melanoma knowledge and beliefs

*Melanoma knowledge* was assessed through 13 items (e.g., “Melanoma is the most common form of skin cancer”) [12, 20, 21]. The number of correct responses created the total score, Cronbach’s alpha (*a*) = .693. Beliefs about melanoma included perceived severity (6 items, *a* = 0.871), perceived controllability (4 items, *α* = 0.669), and perceived risk for melanoma recurrence (4 items, *a* = 0.814), each of which used a 5-point Likert-type scale [12, 23]. *Perceived severity* of melanoma was determined through items such as “How severe would the health consequences of having melanoma again be for you if it was caught early?”. *Perceived controllability* of melanoma was assessed through items such as “Nothing I do will affect my melanoma”. *Perceived risk for melanoma recurrence* was assessed via items such as “I feel very vulnerable to having melanoma again”*.*

##### Psychological measures

*Distress* about melanoma was determined by asking participants to select a number that best describe how distressed they are currently about their melanoma (1 = not at all distressed to 10 = extremely distressed) [12]. *Worry* about melanoma recurrence was assessed through the mean of four items*, α* = 0.918 [22]. An example item assessing *Worry* about melanoma recurrence was “How afraid are you that you may have melanoma again?”. Items for *worry* about melanoma recurrence were measured using a Likert-type scale with 1 = *Not at all afraid* to 6 = *very afraid*.

##### Social influence

Social influence factors included physician recommendation about sun protection behaviors (4 items, *a* = 0.741), descriptive norms regarding sun protection behaviors, and injunctive norms regarding sun protection behaviors [12, 27]. *Physician recommendation* about sun protection behaviors was measured through items such as “Since you were diagnosed with melanoma, has a doctor or other health care professional advised to wear sunscreen with a sun protection factor (SPF) of 30 or more when you are outside on a sunny day?” For each item, participants selected *No* = 0, *Yes* = 1, *I don’t remember* = 2. *Descriptive norms* about sun protection behaviors were determined through the mean of five items (e.g., “My friends and family use sunscreen with a SPF of 30 or more when they are outside on a sunny day”) (5 items, *a* = 0.772). *Injunctive norms* about sun protection behaviors were assessed by the mean of five items (e.g., “My friends and family think I should use sunscreen with a SPF of 30 or more when I am outside on a sunny day”) (5 items, *a* = 0.861). Items for *descriptive* and *injunctive norms* were measured using a Likert scale from *strongly disagree* = 1 to *strongly agree* = 5.

##### Beliefs about sun protection behaviors

These were measured through perceived benefits regarding sun protection behaviors (12 items, *a* = 0.902), perceived barriers regarding sun protection behaviors (23 items, *a* = 0.903), and self-efficacy underlying sun protection behaviors (12 items, *a* = 0.938) [16, 30, 31]. *Benefits* of sun protection behaviors were determined through items such as “Protecting my skin from the sun using sunscreen with a SPF of 30 or more will help me to look younger for longer”. *Barriers* underlying sun protection behaviors were determined through items such as “For me, using sunscreen with a SPF of 30 or more when I am outside on a sunny day is inconvenient”. Items for *benefits* and *barriers* were measured using a Likert scale from *strongly disagree* = 1 to *strongly agree* = 5. *Self-efficacy* underlying sun protection behaviors was assessed through items such as “How confident are you that when you are outside on a sunny day, you can wear sunscreen with a SPF of 30 or more?”. Items for *self-efficacy* were measured using a Likert-type scale *Not at all confident* = 1 to *Very confident* = 5.

### Analyses

Data were analyzed using IBM SPSS Statistics (version 25). Univariate analyses were conducted to assess the relationship between the demographic variables and sun protection behaviors. Hierarchical multiple regression analyses were conducted to evaluate predictors of sun protection behaviors. Variables were entered into the model in seven steps: demographic variables were entered first, then cancer-related factors, skin cancer risk factors, knowledge and beliefs, psychological characteristics, social influence, and beliefs about sun protection behaviors (perceived benefits, perceived barriers, self-efficacy). Change in *R*^*2*^ was computed at each step. Statistical significance was determined at an alpha level of 0.05.

## Results

Table 1 shows the demographic characteristics of the sample. Of participants who were ineligible or declined to participate and provided a reason, the most common were lack of interest, too busy, or no computer access. Ninety-eight percent of the sample was non-Hispanic White. Approximately 68% of the sample were college graduates, and almost 80% were married. On average, patients’ sun protection total score was M = 3.27 (SD = 0.75), with a possible range of 1 to 5. Examination of the four individual items indicated that patients reported using sunscreen, M = 3.83, SD = 1.07, and staying in the shade, M = 3.58, SD = 0.94, more often than they reported wearing either long sleeved shirts, M = 2.84, SD = 1.11, or hats, M = 2.83, SD = 1.34.

**Table 1:** Characteristics of the sample, *n* = 441

Variable	N (%)	M (SD)
**Outcomes** (possible range 1–5)		
Sunscreen ≥ 30 SPF		3.83 (1.07)
Shirt		2.84 (1.11)
Hat		2.83 (1.34)
Shade		3.58 (0.94)
**Demographics**		
Age (range 18–89 years)		61.4 (13.3)
Education		
≤ High school graduate	54 (12.2)	
Some college	87 (19.7)	
Bachelor degree	133 (30.2)	
Graduate degree/professional training	167 (37.9)	
Marital status		
Not married or living with partner	90 (20.4)	
Married or living with partner	351 (79.6)	
Sex		
Female	216 (49.0)	
Male	225 (51.0)	
**Medical factors**		
Months since surgery (possible range 3–24)		14.07 (5.11)
Disease stage		
0	136 (30.8)	
1	245 (55.6)	
2	34 (7.7)	
3	26 (5.9)	
**Skin cancer risk factors**		
Number of risk factors (0–8)		4.35 (1.64)
Sunburn in past year (yes)	114 (25.9)	
**Knowledge** (possible range)		
Knowledge (1–13)		8.04 (2.18)
Severity (1–5)		3.07 (0.84)
Controllability (1–5)		4.02 (0.71)
Risk of recurrence (1–5)		3.24 (0.75)
**Psychological** (possible range)		
Distress about melanoma (1–10)		3.78 (2.25)
Worry about recurrence (1–6)		3.37 (1.27)
Social influence (0–4)		2.63 (1.35)
**Physician influence** (possible range 1–5)		
Descriptive norms		2.63 (0.80)
Injunctive norms		3.61 (0.90)
**Protection beliefs** (possible range 1–5)		
Benefits		4.04 (0.64)
Barriers		2.61 (0.74)
Self-efficacy		3.51 (0.88)

Univariate findings for the demographic variables are reported here. Although there was not a significant sex difference for overall sun protection behaviors, women reported using sunscreen more (women M = 3.99, SD = 0.97, men M = 3.68, SD = 1.15, t(439) = 2.98, *p* = .003) and staying in the shade more than men (women M = 3.68, SD = 0.96, men M = 3.48, SD = 0.92, t(439) = 2.20, *p* = .028). Individuals living with a partner or spouse reported higher average sun protection behaviors, M = 3.31, SD = 0.74, than those who were alone, M = 3.11, SD = 0.78, t(439) = 2.20, p = .028, but there were no significant differences for partnered status on the individual sun protection behavior items. There was evidence of differences as a function of education level. Spitting the sample into two groups (a BA/BS or more education versus those with some college or less), individuals with more education reported higher average sun protection, M = 3.34, SD = 0.75, than those with less education, M = 3.12, SD = 0.73, t(439) = 2.79, *p* = .005. More educated patients also reported wearing a long-sleeved shirt more and wearing a hat more than those with less education (shirt: t(432) = 2.51, *p* = 013, more educated M = 2.93, SD = 1.10, less educated M = 2.65, SD = 1.09; hat t(439) = 2.25, *p* = .025, more educated M = 2.92, SD = 1.34, less educated M = 2.62, SD = 1.32).

Table 2 presents the results of the hierarchical regression model predicting total sun protection behaviors. The demographic variables together accounted for about 3% of the variance in sun protection behaviors. However, the only significant unique demographic predictor over and above all other variables in the model was education level such that more educated individuals (i.e., those with a BA/BS or more education) reported engaging in more sun protection behaviors. Although cancer-related variables did not predict sun protection behaviors, whether the person reported having a sunburn at least once during the past year, together with skin cancer risk factors, accounted for about 4% of the variance. Overall, individuals with greater risk factors tended to report higher sun protection, but those who had had a sunburn reported lower protection. Although neither of these effects were statistically significant in the full model, both were significant in the model that included only those variables along with cancer-related variables and demographics.

**Table 2:** Hierarchical multiple regression results predicting total sun protection behaviors, *n* = 441

	*b*	*95% CI b*	β	*t(419)*	*p*
**Demographics**					
Age	.003	.00 to .01	.054	1.40	.161
Education	.089**	.03 to .15	.111	3.14	.002
Marital status	.049	−.02 to .11	.052	1.50	.134
Sex	−.033	−.09 to .03	−.045	−1.12	.264
			*Step 1* ∆*R*^2^= .032**		
**Cancer-related factors**					
Months since surgery	−.002	−.01 to .01	−.013	−.39	.700
Disease stage	−.044	−.11 to .02	−.045	−1.28	.200
			*Step 2* ∆*R*^2^=.001		
**Skin cancer risk factors**					
Number of risk factors	.029	.00 to .06	.063	1.80	.072
Sunburn in past year	−.113	−.23 to .01	−.066	−1.88	.061
			*Step 3* ∆*R*^2^=.043**		
**Melanoma knowledge and beliefs**					
Knowledge	.009	−.02 to .03	.028	.77	.442
Severity	−.048	−.13 to .03	−.053	−1.16	.246
Controllability	.012	−.06 to .09	.011	.33	.745
Risk of recurrence	.019	−.06 to .09	.019	.51	.612
			*Step 4 R*^2^=.030**		
**Psychological**					
Distress about melanoma	.011	−.02 to .04	.034	.78	.437
Worry about recurrence	.045	−.02 to .11	.077	1.47	.141
			*Step 5* ∆*R*^2^=.018*		
**Social influence**					
Physician influence	.093**	.05 to .13	.167	4.45	.000
Descriptive Norms	.017	−.05 to .09	.019	.50	.614
Injunctive norms	.086*	.02 to .16	.102	2.41	.016
			*Step 6* ∆*R*^2^=.214**		
**Protection Beliefs**					
Benefits	.061	−.03 to .15	.052	1.40	.163
Barriers	−.145**	−.23 to −.06	−.142	−3.43	.001
Self-efficacy	.369**	.29 to .44	.434	9.63	.000
			*Step 7* ∆*R*^2^=.202**		

*Note*. * *p* <. 05, **p < .01. The regression coefficients and t-tests are for the full model that included all predictors. Total *R*^2^ for the model is .539, F(20,420) = 24.56, *p* < .001

The addition of melanoma knowledge and belief related variables increased the percent of variance explained by 3%. Severity, controllability, and risk were not significant in either the full model or the model that included only the previous steps of the model, but melanoma knowledge was a significant positive predictor of sun protection behavior in the model that included only the earlier steps and the four knowledge and belief predictors. People with greater melanoma knowledge reported higher sun protection. Psychological distress and worry added about 2% to the variance explained, but this effect was mostly driven by worry, which was significant in the incremental model but not in the full model.

The final two steps of the model accounted for the highest explained variance in sun protection behavior. People whose physicians encouraged them to engage in sun protection behaviors reported doing so more, and those who reported higher injunctive norms engaged in more sun protection behaviors. This step of the model accounted for 21% of the variance in sun protection behaviors over and above the other variables in the model. The final step in the model, which included variables assessing sun protection beliefs accounted for 20% of the variance. Individuals who reported greater barriers reported lower sun protection behaviors, and those with higher sun protection self-efficacy reported greater sun protection behaviors.

## Discussion

Results from this large statewide sample confirm that engagement in sun safety behaviors among melanoma survivors is suboptimal, particularly the use of protective clothing during sun exposure. In terms of correlates, our finding that sunburn is associated with less sun protection is particularly important. Despite their risk for melanoma recurrence [1], about a quarter of participants reported at least one sunburn in the last year. Some survivors had a sunburn occur after diagnosis or treatment. It is not clear whether these sunburns were obtained due to intentional sunbathing and/or tanning or due to unintentional improper sun protection behaviors. In addition, there are limited available data regarding the level of sun protection needed to prevent sunburns or melanoma recurrence. Regardless, this finding has important implications for intervention.

Survivors who engaged in lower levels of sun protection behaviors had less family and friend support to engage in sun protection. This finding is not surprising, given the importance of social support for many health-related behaviors including those pertaining to cancer prevention and control (e.g., [32, 33]). Although descriptive norms (i.e., family and friends protect their skin) were associated with sun protection behaviors in a prior study of melanoma survivors [12], only injunctive norms were associated with protection in the current study. Perceiving that one’s family and friends think *the survivor* should protect their skin and that their physician recommends skin protection were associated with sun protection behaviors, but the perceived sun protection behaviors of *significant others* was not. This finding is novel. The importance of physician recommendations confirms prior research finding an association with sun safety among melanoma survivors and their first degree relatives [12, 34] as well as other health behaviors such as tobacco cessation and weight loss [35, 36]. However, few randomized controlled trials have examined the impact of physician recommendations on sun safety behaviors [37, 38]. Melanoma knowledge and beliefs overall contributed a small but significant amount of variance to sun protection behaviors. However, contrary to our expectations, perceived controllability of melanoma was not associated with sun protection behaviors, perhaps because participants rated controllability fairly highly with minimal variability, thus limiting the ability to detect potential differences.

Consistent with prior work, survivors with poorer sun protection behaviors were also likely to endorse greater barriers to sun protection behaviors and less confidence in performing these behaviors. Barriers to sun protection behaviors such as perceived inconvenience and unpleasantness of sunscreen, protection clothing, and shade may have contributed to patients’ behaviors leading to their initial melanoma diagnoses and, if ongoing, may contribute to risk for recurrence [1, 3, 39]. Self-efficacy is needed to manage these barriers in order to adopt and maintain sun protection habits [4, 12]. These sun protection beliefs and social influence factors together accounted for 41% of the variance in the sun protection behavior model. Thus, it may be beneficial for healthcare providers and other significant others to recommend sun safety and to assist survivors in developing self-efficacy to address barriers to sun protection, such as by helping to explore options for clothing with high ultraviolet protection factors, various sunscreen formulations, and portable and inexpensive shade structures.

This study offers several implications for future interventions to improve sun safety among melanoma survivors in terms of which behaviors, populations, and psychosocial constructs to target. Interventions for melanoma survivors may benefit from emphasizing wearing protective shirts and hats and avoiding sunburn as well as specifically targeting individuals who have burned in the past or with lower levels of education. Future interventions to improve sun safety among melanoma survivors should also focus on reducing barriers, increasing self-efficacy, and increasing social support, including physician and significant other recommendations for sun protection. For example, prior studies that have combined physician counseling with computerized support that could generate tailored feedback reports have been found to improve sun safety behaviors [38]. A prior intervention study that focused on perceived risk demonstrated improved sun protection behaviors among melanoma survivors [40]. However, perceived risk of recurrence accounted for little variance in our model.

Strengths of the current study include the largest US sample of melanoma survivors from several sources across an entire state and assessment of a diverse set of constructs, including several novel ones. Limitations include the cross-sectional nature of the data and that the sample was highly-educated and drawn only from one US state. Thus, engagement in sun protection may not generalize to other populations and settings. However, the relationships among the correlates and the outcomes are likely similar across populations. Participants in the current study may differ from other melanoma survivors. Although most individuals diagnosed with melanoma are white, our sample was 98% white. Additionally, individuals who reported both high levels of sun protection behaviors and high engagement in skin self-examination were not eligible for the study. However, this accounts for only about 1% of individuals screened for eligibility. Finally, the study did not assess sun avoidance as a potential sun protection strategy, which could have affected the results by lessening the need for other types of sun protection. In conclusion, the current study suggests a need for future research to address psychosocial correlates of sun protection behaviors among high-risk melanoma survivors in order to increase sun safety and ultimately reduce their risk for melanoma recurrence.

## Data Availability

The data that support the findings of this study are available from the corresponding author upon reasonable request.
